# Pregnancy intentions in a group of remote-dwelling Australian Aboriginal women: a qualitative exploration of formation, expression and implications for clinical practice

**DOI:** 10.1186/s12889-019-6925-8

**Published:** 2019-05-14

**Authors:** Emma Griffiths, David Atkinson, Domenica Friello, Julia V. Marley

**Affiliations:** 1 The Rural Clinical School of Western Australia, The University of Western Australia, Broome, WA 6725 Australia; 2 Kimberley Aboriginal Medical Services, Broome, WA 6725 Australia

**Keywords:** Pregnancy intention, Reproductive autonomy, Contraception, Australia, Aboriginal

## Abstract

**Background:**

Unintended pregnancies are associated with poorer obstetric outcomes and are sometimes measured at a population level as a surrogate marker for reproductive autonomy and access to health services, including contraception. Aboriginal Australians face many disparities in health outcomes, including in reproductive health and antenatal care. We aimed to explore the formation and expression of pregnancy intentions in an Aboriginal population to inform health service improvements.

**Methods:**

Semi-structured interviews were conducted with 27 remote-dwelling Aboriginal women, aged 18–49 years. Content analysis was conducted; key themes were discussed with groups of women from participating communities to refine interpretation.

**Results:**

Most (19/27) participants expressed pregnancy intentions congruent with reported contraceptive behaviour while eight expressed ambivalent or uncertain intentions. Intentions were shaped by traditional kinship practices, reproductive autonomy and desired family formation. Younger women tended to aspire to smaller family sizes than older women and support was expressed for the postponement of first pregnancy to achieve other life goals. Women in these communities hold strong traditional beliefs, including regarding conception, but did not use traditional methods of contraception in place of modern methods. Reproductive coercion, in the form of pressure to fall pregnant, was recognised as an important issue by women in the community.

**Conclusion:**

Consultation strategies that promote rapport, allow space for uncertainty and are inclusive of important personal and cultural contexts are likely to improve shared understanding of pregnancy intention. Universal screening for reproductive coercion and broad counselling on contraceptive options (including discrete methods) may reduce unmet need for contraception. Community approaches supporting reproductive autonomy that is inclusive of men, and enhanced educational and occupational opportunities for young women are needed.

## Background

Unintended pregnancies (often further classified as unwanted or mistimed) [1] have been associated with later entry into antenatal care, increased frequency of pregnancy risk factors and adverse pregnancy outcomes [2]. A third of pregnancies in Australia are estimated to be unintended [3]. At a population level, the rate of unintended pregnancy is sometimes used as a surrogate marker for women’s reproductive autonomy and access to health services [4]. At an individual level, assessment of pregnancy intention is important for the provision of appropriate contraceptive services and timely preconception care.

For these reasons pregnancy intentions and the association of intention with relevant actions (such as contraceptive use, adoption of healthy pre-conception behaviours and early access to antenatal care) are concepts fundamental to the understanding of reproductive health issues and the provision of quality care. Discrepancies between reported pregnancy intentions and pregnancy related actions have been well demonstrated in the literature [5–7]. This prompts questions around whether methods of assessing pregnancy intention are accurate, and what contextual factors impact on the relationships between intention and actions. Pregnancy intention and associated actions may be shaped by factors such as the degree of female reproductive autonomy, socioeconomic status, pro-natalist norms and attitudes and values towards available contraceptive options [8]. Traditional theories of reasoned action have been challenged in this space [9], highlighting the need for individual consideration of planning, intention, desire and action when discussing pregnancy intention.

It has also been suggested that women may fail to form pregnancy intentions because of low perceived reproductive control [6]. The concept of “planning” has been described as an “unattainable ideal” in qualitative studies of populations of disenfranchised women [6]. This is likely to present the greatest barrier when the act of planning is defined by the attainment of life goals that seem irrelevant or unlikely, or when planning requires foresight into the distant future. The process of assessing pregnancy intention often implicitly assumes a high level of reproductive autonomy and agency within partnerships. However, pregnancy intentions within couples is not always concordant, requiring a degree of negotiation. On one end of the spectrum this may affect the formation and expression of pregnancy intention via reproductive coercion. Defined as behaviour that interferes with the autonomous decision-making of a woman with regards to reproductive health [10], reproductive coercion has been described in many different populations [6], and has an association with other forms of intimate partner violence and with unintended pregnancy [10].

Australia is a developed country with an efficient health care system that provides superior health outcomes in comparison to other Commonwealth countries [11]. Aboriginal Australians, however, have one of the longest living cultures yet drastically poorer health outcomes than their non-Indigenous counterparts [12]. National targets to reduce inequities in health outcomes are currently not on track [13] and complacency on this issue cannot be afforded.

In Aboriginal Australian communities culturally-specific traditional kinship practices are historically integral to the determination of partnerships and family formation. Kinship-based marriage organises all social structures and relationships, determines obligations to land and family and imparts identity within and between social groups. Disruption to the kinship system has the potential to impact across communities in terms of relationships, ritual practices and marriage eligibility in current and subsequent generations, as illustrated in historical accounts of forced remarriages in missionary times [14]. Additionally, young people’s desire for autonomy has been described as increasingly competing with traditional marriage practices in influencing partnering and pregnancies [15].

Our previous work examined the uptake, continuation and acceptability of contraception methods in three remote Aboriginal communities [16]. The most common form of contraception used in these communities is the etonogestrel implant and continuation rates with this method were good. Thematic analysis of interviews with women demonstrated good acceptability of method, with adverse effects being troublesome for a subset: four of 17 women who had used the implant had ceased due to unwanted side effects. However, despite this apparent acceptability, incident contraception use was documented for only one-fifth of women in these communities, compared with two-thirds of all Australian women aged 18–49 years in 1998 [17]. Also, some women described pressure from partners to cease contraception. This raises the possibility of some unmet need for reproductive health services: to better understand what needs might be “unmet”, we must first better understand those needs.

In this article we present a further exploration of the formation of pregnancy intention through analysis of semi-structured interviews with Aboriginal women residing in participating Western Desert communities. In improving our awareness of the expression of these intentions and the factors that influence them we aim to develop a shared understanding of their reproductive health needs in order to enable improvements in health services.

## Methods

### Recruitment

This study involved the continuation of a mixed method design focussing on contraception use and acceptability. Quantitative data relating to contraceptive use and continuation has been previously published elsewhere [16]. Women were eligible to participate if they identified as Aboriginal and/or Torres Strait Islander and were of adult reproductive age, defined as 18–50 years inclusive. No women were knowingly pregnant at the time of interview. Consent was sought at the time of interview. Purposeful sampling was used to recruit women who indicated an active interest in discussing this topic and to ensure different ages and stages of reproductive life were included. Interested women were identified through existing community relationships and through word of mouth from participants. Light refreshments were provided during and after interviews for participating women. Local female Aboriginal Health Workers were offered an opportunity to provide individual interviews or contribute to feedback and analysis according to their individual preferences.

### Setting

The participants were from three very remote communities in the Western Australian (WA) Western Desert region with a combined population of 915 at the 2011 census [18]. The two smaller communities are located within 90 min’ drive of the larger community. A general practitioner resides in the largest community and provides medical services to all three. The current primary health provider organisation has been providing health services to the region since 2006.

Prior to colonisation the lake system in the region was a centre of activity lying at the end of a dreaming track that brought together many language groups over a wide area. The area has a history of occupation by Catholic missionaries since the early 1940s who provided health and other services during the post-war era. In 1983 the mission handed over its land and administration, and the communities became self-managed [19]. The current communities include families from multiple language groups from the surrounding desert. A local Catholic school has provided education to year 9 level since 1984, after which young people may travel away from their community to complete high school. Although many people speak English well, it is often as a second or third language. “Bush medicine” and traditional healing practices are understood and continue to be practiced, sometimes in parallel with Western medicine.

The participating communities continue to observe a separation of “men’s business” from “women’s business”, both in traditional law and cultural practices and in discussion of reproductive health issues. A traditional practice of “promised marriage” is well documented where young women were betrothed at a young age to an older man, however this has been noted as becoming less common over time [20]. Kinship structures are also still prominently recognised and dictate the acceptability of certain social interactions. One important aspect of kinship are the sets of “skin names” used in the region which can vary by language group. Skin names of children are determined by their ancestry and in traditional societies determine aspects of most social interactions, including who a person is permitted to marry and also who they are expected to avoid. The phrase “wrong skin” implies a relationship that would have traditionally been prevented by the kinship system [21]. This system allows individuals across multiple generations to understand their relatedness and for this knowledge to be passed down through subsequent generations, ensures a degree of genealogical distance and provides continuity in relationships with the land and other population groups [22]. Disruption of traditional marriage systems can result in confused and contradictory relationship definitions between two people [14]. These disruptions have ripple effects through subsequent generations, particularly in combination with other community fragmentation such as forced removals and decreased sex segregation.

### Reflexivity

Interviews were conducted by the sexual health nurse who had been based in these communities long-term (author DF) and a doctor who had visited the community over several years (author EG), both female and non-Indigenous.

The authors acknowledge the privileged position occupied in this space as clinician researchers, and the potential for conflict in this dual-role [23]. Where indicated, arrangements were made to follow-up the clinical needs of participants after the completion of the interview. We also note the limitations inherent in our role as non-Aboriginal service providers: trusted and known as long term service providers, but external to the community and therefore outsiders. Our process aimed to create a safe environment for cross-cultural exchange and knowledge transfer that reduced the distance between key community informants and research end-users.

### Interviews

Questions were drafted and piloted with Aboriginal colleagues. Visual aids were used which included interview questions in plain language and large font, Likert-type scales and categorical options. Interviews, which ranged in duration from 20 to 40 min, were conducted in a clinic consulting room after hours, or a vacant community building, based on participant preference and room availability. Interviews were conducted in English. Women were invited to bring support persons to the interview with them, however all interviewed women elected for private interview. Question content was revised iteratively incorporating initial analysis of responses and direct feedback from participants during interviews. Three women were interviewed a second time, following a revision of the interview guide. Question content is summarised in Table 1.

**Table 1:** Content of semi-structured interviews

Category	Content	Questions included in the prompt sheet^a^
Contraception	Contraception used; Reasons for use; Experiences of using;	Are you using any [contraception] at the moment? Have you used any/any other [methods] before? What is good/bad about this/these [methods]? Have you heard of any other [methods]?
Pregnancy intention	Wanting, trying; Timing; Family size; Hypothetical pregnancy;	Do you want any [more] babies? Right now, are you trying to / trying not to have a baby? How long between now and when you want to have a baby? How many babies would you like to have all together? If you found out you were pregnant today, how would you feel? What would you do?
Decision-making	Choosing a partner; Marriage; Age and pregnancy;	How do you decide a man is the right one to have a baby with? Is it important to be married before having a baby? How do you decide it is the right time to have a baby? What is a good age to start / stop having babies?
Partner	Wanting; Timing; Family size; Talking;	Does your partner want any [more] babies? How long between now and when your partner wants to have a baby? Do you and your partner have any talks about having babies?
Autonomy	Personal experiences of; Perceptions of issue in community;	Has anyone ever tried to force you / pressure you about: contraception/having a baby/ending a pregnancy? Is forcing / pressure an issue in [this community]?

^a^Exact wording was modified as needed to enhance comprehension. Important points were clarified by revisiting questions with alternative phrasing to improve the accuracy of interpretation

Initially interviews were hand transcribed at the time of interview by one interviewer and cross-checked with the second interviewer within 48 h. Based on feedback from women during the course of the project that audio recording would be acceptable to the community, an ethics amendment was submitted to allow audio recording as an option. All subsequent participants agreed to audio recording – these recordings were subsequently transcribed by one interviewer and cross-checked between interviewers within a week of interview.

### Analysis and feedback

The results of an initial analysis of these interviews were presented back to focus groups in two of the three communities (open to any interested women) using plain language summaries to stimulate discussion and to test our interpretations of the data. Feedback from local women was then used to inform further analysis and prioritisation of themes. Content containing culturally specific knowledge was de-identified then discussed with a cultural custodian identified by the community; specific knowledge that was considered important Aboriginal cultural property has been excluded from this publication.

Responses relating to pregnancy intention in the short and long term, desired family size and contraception plan were used to summarise pregnancy intention and were analysed for congruency. Explanatory factors relating to intention formation and decision making around conception and contraception were coded for thematic analysis. Analysis sought to address the following research questions: Are women forming pregnancy intentions? What influences the formation, or lack of formation of pregnancy intentions? Are unmet needs for contraception revealed through exploration of pregnancy intentions?

## Ethics approval

Aboriginal women elders from the communities were initially consulted during the development of the project, discussed the project and agreed that the project was consistent with local priorities. This project received ethics approval from the Western Australian Aboriginal Health Ethics Committee (reference, 585) and was supported by the Kimberley Aboriginal Health Planning Forum Research Subcommittee.

## Results

The final sample included 27 women, who ranged in age from 18 to 49 (median 28) years at time of interview. Women’s prior births (parity) ranged from zero to five (median 2). Participant characteristics are summarised in Table 2.

**Table 2:** Participant characteristics

Characteristic	Group	Number of participants
Age	18–24	10
	25–34	8
	35–44	8
	45–49	1
Parity	0	7
	1	4
	2	5
	3	4
	4	5
	5	2
Contraception in situ at time of interview	Contraceptive implant	12
	Permanent (tubal ligation or hysterectomy)	5
	Medroxyprogesterone depot	2
	Nil	8
Pregnancy intention	Current positive	7
	Future positive	5
	Negative	7
	Ambivalent/uncertain	8

### Pregnancy intention

The majority of women (*n* = 19) expressed congruent pregnancy and contraceptive intention, with either current positive (“yes, now”) (*n* = 7), future positive (“yes, but not yet”) (*n* = 5), or negative (“no more”) (*n* = 7) intention.

Women who had congruent present positive pregnancy intention were not using contraception, would be “very happy” to be pregnant, were “trying” to become pregnant and described an ideal family size that was greater than their current number of children. Women who had future positive pregnancy intention had contraception in situ or a plan to access contraception and expressed positive intentions, but for the “future”, or “later on”.

Women who had congruent present negative pregnancy intention had contraception in place or a plan to access it and reported “trying hard not to have a baby” or being satisfied with their current family size. These seven women had at least one previous pregnancy. In contrast, eight women expressed some ambivalence or uncertainty regarding the possibility of future pregnancy. Some women were hesitant to disclose their positive pregnancy intention until the later stages of the interview as greater comfort and rapport was achieved. Time-specific questions were necessary to ensure a mutual understanding of timing with respect to pregnancy intention:

INT: Do you want any babies do you think?

P11: Yeah but I think... ah... I need a good a man sometimes I think.

INT: How long between now and when you want a baby?

P11: Like wait when I get older, a little bit older.

INT: How much older is older?

P11: One month.

In discussing the future women tended to only discuss time periods of months, or perhaps “future” to indicate more distant, less able to be predicted, possibilities. Whether or not they desired a pregnancy, most women clearly stated that if they found out they were pregnant they would have the baby and raise it themselves, in preference to adoption or termination of pregnancy.

### Finding a partner, the “right way”

Traditional kinship structures remain important to women in this community. Women had strong feelings that there were “right ways” and “wrong ways” to form partnerships. Personal attributes such as being hardworking, fit, strong, and treating women well were thought to be important. For some, marriage was important. One woman had memories of participating in her parents’ marriage in the local church (marrying “Kartiya [non-Indigenous] way”). However, for most women marriage referred to the traditional practice of Aboriginal law and kinship structures.

Marriage itself was not always viewed as important if the partnership in question was well known and accepted by the community, hence legitimate (P21: “They got baby and they not married, he still the family. The family will know that they were going out nearly every night”). For some their views reflected their own experiences (P23: “It was my choice, I was very young and didn’t want to be married”). One woman had experience of being in a “promised” marriage. Some expressed that unlike other aspects of kinship-marriage, a large age gap between husband and wife was no longer acceptable, and that it was important to know a man before marriage.

P21: Sometimes they just married “blind way”. They should know them first better, they should know the person better, they should know the persons family and know him better.

Being “wrong skin” or having certain other relationships under Aboriginal law was an important reason that some people could not marry and should not have a baby together. These “wrong way” partnerships were frowned upon as they were considered to lead to uncertainty about the paternity of babies, community fighting and unrest.

P22: When they have a partner, then they can have a baby. It’s not right when they have no partner but they be pregnant, cause trouble like that. Like the other man says ‘my baby’, then the other man says ‘that’s my baby’. That thing cause more problem… there was fighting a couple of months ago about baby.

Kinship systems were also seen to impact on family structures and bringing up of the child.

P25: Here's what I was doing, I didn't want [my baby’s] dad involved because he was with another woman, I kept my baby to myself.

INT: Could he have said to you, ‘But I want to hold the baby. I want to grow it up’?

P25: Nup, he know it was wrong skin and ... yeah. Yeah, if it was right skin I would have given [the baby] to him, but wrong skin and woman, I kept my baby for myself.

In contrast to this, babies born to “right skin” partnerships were seen to bring happiness and harmony to the community:

P25: … But it brings family together, in-laws to in-laws, together. Sometimes the baby... yeah, they're good family like that you know... but we in the community see, little communities, like it brings people together [but] sometimes especially if they're wrong skin it doesn't work. If they're right skin it brings family together.

Women in insecure relationships were judged for “mucking around” and not knowing who they had been with (P22: “Because they don’t know which man they going out with… they go one, one day, another the next day”). Five participants discussed this as an important issue and held these women responsible for fighting in the community. It was believed that a pregnancy had a changing effect on both mother and father, and men were sometimes scrutinised to see if the pregnancy was “making them different” (P27: “Sometimes when they, like when the partners not getting different, and they see that man is getting… you know... different, getting weight or losing weight”). Where paternity was disputed, a woman was at risk of getting caught up in fighting between family groups.

### Making a family: timing, size and gender: “I’d be happy, if I had another little baby girl”

Women recognised the benefit in postponing pregnancy until a certain age; for most women this was 18–25 years. One reason was to give women time to achieve other life goals (P8: “I reckon they should go to Perth, finish school, go to university, get good jobs, then have babies”, P20: “Probably when you are all organised, got money, got car”). Most women identified the ideal age to fall pregnant as older than they had been at their first pregnancy. Education was described as something that would complement motherhood:

INT: How old is old enough?

P21: I dunno, well only times you can have babies is when she finished schooling, [going to] college, maybe after that she can do whatever she wants, because she’ll be well educated now.

INT: Is it important to be well educated?

P21: Yes. So if kids bring their things [school-work] to you the mum can do it. Otherwise they come to her [for help], like asking ‘Mum, help me read this’ and she’ll be like ‘Go ask your father, your grandmother’, and say ‘I’m too tired’ but she is lying because she doesn’t know how to read.

The other reason to postpone pregnancy was the concern that pregnancies at a young age would be dangerous for the young woman (P27: “…for me it’s not alright for young girls, like young teenager to have a baby because you know, sometimes they get killed from babies you know…”). It was felt that if parents did not discuss contraception with young teenagers, they might be at risk of an early pregnancy.

Women identified the need to space pregnancies in order to be well placed to manage a family (P22: “Wait ‘til baby get big, walking maybe before you have another. Can’t have another baby while my baby is still small”). Desired family size ranged from one to seven. Most women aged under 25 (*n* = 6) reported a preferred family size of one or two children (5 of 6), whereas women aged 30 years and over (*n* = 15) had ideal family sizes of three (3 of 15), four (7 of 15) or more (3 of 15) children. Women reflected on the practicalities of caring for multiple children at once and the potential for stress:

P27: When some womans’ got five or six… like she got…. Baby, sitting down baby and she got a maybe two, three or five years old… you know she comes and cries at the mother’s lap ‘Ahhh’ like you know, the mother goes mad and starts giving her a hiding.

INT: And why do you think she does that?

P27: Don’t know… I think she got stressed or something like that, stressed from too many kids… Wait until she or he is maybe walking around, walking, walking baby… Because you can’t handle it like, when he or she is still a baby and you want to have another baby, you know, it’s going to be rushed like…Too hard.

For some women the desire to have a girl, or another girl was central to their desire for additional pregnancies (P23: “I’d be happy, if I had another little baby girl”). Girls were seen as more helpful to the mother, easier to manage and to have a role in caring for younger children, and it was expected that one daughter would want the companionship of a sister to grow up with (“P5: One girl grows up, want more sister, she’s going to ask me for that”.

### Autonomy and control

Pressure from partners to fall pregnant was perceived as a common problem. Although mentioned by nine participants, it was largely discussed at a distance, happening to “friends” and “other women”. This occurred through pressure to cease contraception and the threat that men would leave women who did not fall pregnant. One woman described switching from the contraceptive implant to the medroxyprogesterone injection so she could access contraception in secret. This was normalised by some women, as an expected part of gender dynamics.

P24: But I been hear some girls, like maybe, maybe boyfriend one was telling them have baby, and they go tell me… I dunno, maybe just have it... make the boy happy, and the family.

INT: Is that scary for girls sometimes?

P24: No... they just feel happy, like fun. They just talk ‘Ahh, can you take that implant off, you gotta have my baby’, like that. And the girl just laugh ‘Yeah I'll take it off, I'll go see the doctor’.

INT: What if the girl says no, what will he do?

P24: Well, you won't meet him again.

The solution was sometimes to go “quietly” to the clinic and get their contraceptive implant removed, suggesting that reproductive coercion, if present, was not always openly discussed with clinic staff.

P27: Quietly for him. To make him stop having argument. Sometimes, men get wild, because the girl got that implant on and [she’s] not allowed to have… you know, private... private, some of them have their private problem, you know, they don't tell us maybe…

For one woman, her future pregnancy plans were uncertain in anticipation of pressure to become pregnant.

P8: My partner just came out of prison…. but I not ready yet... Some women stay strong, keep their [implant] in. I been through that with my partner, try to tell him I’m not ready, he not support me. Some young girls, some girls say no… Three is enough I think I just keep this [implant] in.

Women expressed frustration at being on the receiving end of jealousy. They attributed some problems to men’s lack of understanding about the inheritance of traits or the timing of conception.

P21: He [her oldest child] has my mother’s skin colour. He’s got my mother’s bright skin colour… but the baby is for [her current partner], but the colour comes from mother side of family. He keeps saying ‘Only [the youngest child] is mine’.

P23: Maybe [when she gets pregnant], he’s gone out, he might come back and say ‘I’ve gone out, this is someone else’s’. Men can ask silly question you know… If the woman got pregnancy and men don’t know nothing she’s already expecting a baby and it’s still growing and a couple of months later they see she’s getting more bigger, ‘Is that mine or somebody else’s’?

For most women, conversations with their partner about pregnancy planning were brief or did not occur. Women were not always certain why men might want to have babies, suggesting that he would take pride in being a father (P21: “because he wants to take it around and show it to family, and they congratulate him”), or that it will make him stronger and happy.

### Western versus traditional beliefs

Most women described traditional fertility practices as having an important role, particularly in becoming pregnant (details omitted at community request). Despite these strong connections to traditional practices, women also discussed the importance of “Western” medicine and talked about the experiences they had had accessing clinic services. Participants identified that when pregnant, it was important to go to the clinic to confirm the pregnancy and have a check-up, however they also suggested that in some cases a woman might hide her pregnancy intention to avoid “humbug” from the clinic during pregnancy, in order to conceal a “wrong skin” relationship, or because she was worried that she might not fall pregnant quickly. Infertility was associated with sadness and shame, and participants were uncertain about why some women were unable to get pregnant.

P26: Some trying to have babies... but couldn't… they married right. Some young girls you know but they can't have babies.

INT: Do you know why it is that they can't have a baby?

P26: I don't know.

One woman, however was interested in attending the clinic for assistance with fertility (P25: “Nah I want to find out about [how to] have a baby, take pills or anything… medicine to make baby come”). Women also identified getting a check-up for “STI” or “germs” as a good reason to go to the clinic, to avoid “damage… like sore eyes (for the baby) … [and to check you are] healthy and you know, not sick” (P24) and also “iron tablets, make strong baby (P5)”.

## Discussion

Our study confirms that it is possible for clinicians to develop a shared understanding of pregnancy intention for most women in this community. Participating women who wanted to avoid pregnancy were generally accessing modern contraceptive measures. Some women did not express clear pregnancy intentions or were uncertain about future pregnancy plans and these women appeared to have lower perceived reproductive control.

Women spoke of the continuation of traditional cultural practices in parallel with the use of Western medicine. Partnering and the formation of pregnancy intention in this population remains closely associated with traditional kinship systems and women generally have a clear understanding of, and attachment to, traditional fertility practices regarding conception. However, women concurrently described the importance of using Western medicine to ensure a healthy pregnancy with a good outcome. No woman reported a preference for Aboriginal traditional methods of contraception. One published reference to “bush medicine” for contraception on Groote Eylandt tells of the use of herbs to impart permanent contraception [24], but such reports are rare. In this study we did not detect unmet need for modern contraception on the basis of preference for traditional or other alternative methods.

The interview responses suggested a decline in ideal family size in association with older preferred age at first pregnancy and support for the education of women. This finding was significantly different from attitudes and values documented in a 1987 study, where the use of contraception to delay a first pregnancy was frowned upon in a number of Aboriginal communities across Australia [25]. Expressed interest in education also appears to be higher than in an earlier study with young Aboriginal women in 2003 [26]. Such changes may well be suggestive of demographic transition [27], although traditional demographic theories may have limited applicability to collectivist cultures and the complexities of the post-colonial context [28].

Women in our study idealised the notion of further education and financial security, even though this was achieved by few women from these communities. Currently this would require young women to make the difficult choice to travel far from country and family in order to pursue education and employment. Although rates of teenage pregnancy are declining for Aboriginal women, the national dataset still shows rates to be more than six-times higher than in the non-Indigenous Australian population [29]. Supporting young Aboriginal women with educational and occupational opportunities is essential in allowing them to actualise their life goals.

Reproductive coercion was an issue of concern for women in these communities, mostly consisting of pressure to become pregnant. This is also an international health concern, with women from disadvantaged or disempowered groups being disproportionately affected [10]. Studies in the clinic environment have identified value in informing women about “hidden” forms of contraception [30] and randomised-controlled trial data demonstrates that universal screening for reproductive coercion can be well accepted [31]. Increased self-efficacy and awareness of support resources can reduce reproductive coercion for some women [32]. However, these approaches place the burden of change on women who are already disempowered and lacking control in their life.

Approaches that are inclusive of the partner are important and can be effective in reducing intimate partner violence and enhancing reproductive autonomy [33, 34]. A recent statement from a Kimberley Aboriginal Men’s Health Gathering has further highlighted the importance of including men in family and early childhood initiatives and emphasised the importance of the role of Aboriginal men as fathers [35]. It is likely that sustained improvements to reproductive autonomy will require a reorienting of community attitudes and norms over time, which will hopefully be enhanced by new community programs addressing family violence more generally. Approaches such as working with men, encouraging community healing and “country-centred” responses were identified as important in a recent Australia’s National Research Organisation for Women’s Safety report on addressing violence against Aboriginal and Torres Strait Islander women [36]. Our data suggest that men may also benefit from the delivery of culturally appropriate education about topics such as normal pregnancy and intra-uterine development, when a pregnancy begins to “show” and how physical traits are inherited as part of more general education about antenatal care. This may help remove triggers for arguments between couples that are based on misunderstandings.

Assessing pregnancy intention in a one-on-one clinical encounter is an important prerequisite to the provision of information on available preconception and contraception options. Some women only disclosed their desire for pregnancy after a rapport had been achieved. We noted a tendency for congruity of expressions of intention to increase (or alternatively, for our understanding of intentions to improve) as the interviews progressed and women expanded on relevant contextual factors. This required time and gentle inquiry, exploration of cognitive and affective aspects, the use of time-specific questions, and the discussion of a hypothetical pregnancy. Despite best efforts, achieving a shared understanding of pregnancy intention across cultures can be difficult. Women who intend a pregnancy with a man deemed unsuitable by their community, or who experience reproductive coercion may be reluctant to discuss their situation, even in health care consultations bound by confidentiality.

An approach to questioning which allows for (and does not invalidate) ambivalence and cognitive dissonance has been advocated [4]. We believe this is important for clinicians delivering remote area health services to Aboriginal people, and for clinicians working in reproductive health more broadly. This approach allows the health care practitioner to provide information and counselling on the full range of options available to a woman (including “hidden” methods of contraception), without imposing a forced dichotomy or further marginalising disempowered women. It has been suggested [6] that being prepared “for whatever might happen” may be conceptually more appropriate than “planning” for women who either lack reproductive autonomy or who express uncertainty, incongruence or ambivalence regarding possible future pregnancies.

One important limitation of our study is that we as clinicians were poorly placed to explore women’s perceptions of autonomy within the clinic. This may warrant additional study as providers are likely to bring their own expectations of ‘normative readiness’ to contraceptive consultations [37]. Studies have shown a tendency of health care providers to discourage LARC discontinuation when patients present with adverse effects or when the provider judges them “not ready” for a pregnancy [38–40]. Also relevant is our lack of a male perspective from the participating communities. This represents a significant gap in the literature on pregnancy intention and reproductive autonomy more generally. Women in our study were reluctant to guess at men’s motivations, and since procreation, conception and pregnancy are highly gendered topics in this community we as female interviewers did not attempt to address this subject directly with local men.

Despite our efforts, our selection of participants would tend to bias towards the inclusion of women who were more comfortable speaking English, more confident addressing clinicians, and who may be more empowered in other ways. English would likely be a second-language for most women. Women preferred to speak to us individually, without other community members, and we were not able to offer an unrelated (from out of community) translator which some women might have found useful. Continuing support and empowerment of Aboriginal researchers to navigate such issues would be important in addressing this frequently challenging issue. Finally, this was a qualitative study rather than a quantification of unmet need, and specific to the participating population, which may not reflect what is happening in other populations.

Further studies with male researchers and Aboriginal men are required to facilitate a balanced understanding of this issue and to inform community men’s health programs. Another area that may need further exploration is the nature of understanding and health beliefs related to infertility, which was clearly very important to women, but poorly understood by them. This is a population where high rates of risk factors for infertility (including sexually transmissible infections and metabolic syndrome) [12] intersects with strongly held beliefs regarding the origins of conception.

## Conclusions

Our study provides clinicians with valuable input from women that could be used to inform improvements to the delivery of culturally safe reproductive health services, and highlights areas where further research is needed. At a community level, strategies to increase women’s autonomy, educational and occupational opportunities for young women and appropriately designed and implemented men’s health programs could help prevent reproductive coercion and enable improved access to reproductive health services. Equally, in women who do desire a pregnancy, pre-conception care delivery may be enhanced by supporting providers to assess pregnancy intention in ways that are respectful and inclusive of important personal and cultural contexts. We have begun incorporating these results into additional regional guidelines and provider resources to support reproductive health consultations and the delivery of preconception care. These are global health priorities as women of First Nations or postcolonial populations in many countries are required to seek assistance from health providers from different backgrounds. This paper describes approaches that could be used in other countries facing these challenges in health services provision.

## Abbreviations

LARC: Long-Acting Reversible Contraception
WA: Western Australia
